# Ascorbic acid mitigation of water stress-inhibition of root growth in association with oxidative defense in tall fescue (*Festuca arundinacea* Schreb.)

**DOI:** 10.3389/fpls.2015.00807

**Published:** 2015-10-02

**Authors:** Yi Xu, Qian Xu, Bingru Huang

**Affiliations:** ^1^Department of Plant Biology and Pathology, Rutgers UniversityNew Brunswick, NJ, USA; ^2^National Engineering Laboratory for Tree Breeding, Beijing Forestry UniversityBeijing, China

**Keywords:** turfgrass, water stress, root growth, reactive oxygen species, cell wall, gene expression

## Abstract

Root growth inhibition by water stress may be related to oxidative damages. The objectives of this study were to determine whether exogenous application of ascorbic acid (ASA) could mitigate root growth decline due to water stress and whether ASA effects on root growth could be regulated through activating non-enzymatic or enzymatic antioxidant systems in perennial grass species. Tall fescue (*Festuca arundinacea* Schreb. cv. “K-31”) plants were grown in nutrient solution, and polyethylene glycol (PEG)-8000 was added into the solution to induce water stress. For exogenous ASA treatment, ASA (5 mM) was added into the solution with or without PEG-8000. Plants treated with ASA under water stress showed significantly increased root growth rate, and those roots had significantly lower content of reactive oxygen species (ROS) (H_2_O_2_ and O${}_{2}^{-}$ content) than those without ASA treatment. Malondialdehyde content in root tips treated with ASA under water stress was also significantly reduced compared with those under water stress alone. In addition, free ascorbate and total ascorbate content were significantly higher in roots treated with ASA under water stress than those without ASA treatment. The enzymatic activities for ROS scavenging-related genes were not significantly altered by ASA treatment under water stress, while transcript abundances of genes encoding superoxide dismutase, catalase, ascorbate peroxidase, glutathione reductase, dehydroascorbate reductase, and monohydroascorbate reductase showed significant decreases in the root elongation zone and significant increases in the root maturation zone treated with ASA under water stress. Transcripts of genes for expansins and xyloglucan endotransglycosylases showed increased abundances in ASA-treated root maturation zone under water stress, indicating that ASA could accelerated cell wall loosening and cell expansion. The results suggested that exogenous treatment of roots with ASA enhanced root elongation under water stress, which could be attributed by increasing non-enzymatic antioxidant production, suppressing ROS toxicity and up-regulating gene expression of cell-wall loosening proteins controlling cell expansion.

## Introduction

Cell growth is one of the most sensitive processes to water stress, with a restriction of cell expansion occurring at a water potential of −0.5 MPa (Nilsen and Orcutt, 1996). Therefore, water stress is one of major limiting factors for root growth (Morgan, 1984; Sharp et al., 2004; Bengough et al., 2011). Maintaining active root growth is therefore critically important for plants efficiently utilizing water under water stress conditions.

Many metabolic factors could contribute to the inhibitory effects of water stress on root growth (Chaves et al., 2003). It is well known that water stress induces the production of reactive oxygen species (ROS), such as superoxide (O${}_{2}^{-}$) and hydrogen peroxide (H_2_O_2_), causing oxidative damages, which could be related to root growth restriction due to water stress (Wu et al., 2006; Bian and Jiang, 2009; Selote and Khanna-Chopra, 2010). Plants develop non-enzymatic antioxidants and the enzymatic scavenging systems to detoxifying ROS (Mittler, 2002; Mittler et al., 2004). In the enzymatic pathways, the damaging O${}_{2}^{-}$ produced from electron transfer chain would be first catalyzed by superoxide dismutase (SOD) into H_2_O_2_. The H_2_O_2_ is further reduced into water by catalase (CAT), peroxidase (POD), or by ascorbate peroxidase (APX), glutathione reductase (GR), monodehydroascorbate reductase (MR), and dehydroascorbate reductase (DR) into H_2_O. Non-enzymatic antioxidant compounds, including ascorbate (ASA), possess intrinsic antioxidant properties, serving as electron donors to directly reduce ROS accumulation (Noctor and Foyer, 1998) and also as reaction substrates within the enzymatic cycle (Mittler, 2002). In addition, ASA also play roles in stress signaling and other physiological processes (Smirnoff, 1996; Wolucka et al., 2005). The accumulation of free ASA in plant cells is of great importance for ROS scavenging since it is involved in both non-enzymatic and enzymatic systems, as well as the other important functions. The physiological function of ascorbic acid under water deficit stress has been studied in leaf tissues (Al-Hakimi and Hamada, 2001; Shalata and Neumann, 2001; Athar et al., 2008; Beltagi, 2008; Dolatabadian and Saleh Jouneghani, 2009; Dolatabadian et al., 2009), and in roots (Al-Hakimi and Hamada, 2001; Shalata and Neumann, 2001; Afzal et al., 2006). Moreover, the *Arabidopsis* ascorbate-deficient mutants were also obtained to study the physiological role of ASA *in vivo* (Conklin et al., 1996, 1999; Huang et al., 2005). The benefits of exogenous ASA application in order to attenuate water deficit stress include increased nutrient uptake, improved leaf and root growth, reduced lipid peroxidation, and relieved oxidative stress (Khan et al., 2011). A lot of efforts have been taken into the study of applying ASA to foliar part of plant in order to promote shoot growth under environmental stresses (Khan et al., 2006; Farahat et al., 2007; Dolatabadian et al., 2008, 2009). However, there is little information available to link ASA and ROS scavenging with its impact on root growth under water stress conditions.

Cell expansion is controlled by cell-wall loosening proteins that modify the linkage of cellulose microfibrils and thus cellular matrix, such as expansin (EXP) and xyloglucan endotransglycosylase (XET) (Cosgrove, 2000). The expansins is a gene family that contains multiple gene members, mainly grouped as α-, α-like, β- and β-like expansins, which share conserved protein domains (Li et al., 2003; Choi et al., 2008). Expansins are believed to bind to the surface of cell wall microfibrils and disrupt hydrogen bonds between cellulose microfibrils and crosslinking glycans, although lacking hydrolytic activity (Cosgrove, 2000, 2005; Li et al., 2003). XETs also belong to a class of cell wall enzymes that are responsible for the cleavage of xyloglucan chains and the reconnection of their reducing ends to non-reducing ends, thus enable microfibril relaxation and cell expansion (Fry et al., 1992; Rose and Bennett, 1999). Evidences have been found in both expansins (Yang et al., 2004; Buchanan et al., 2005; Sabirzhanova et al., 2005; Li et al., 2011) and XETs (Xu et al., 1996; Bray, 2004; Cho et al., 2006) that they are also induced by drought stress. However, whether ASA-mediated root growth responses to water stress is associated with expansin and XET genes remains unknown.

The objectives of this study were to determine whether treatment of roots with ASA could mitigate root growth decline due to water stress and whether ASA effects on root growth could be regulated through activating non-enzymatic or enzymatic antioxidant systems and changes in gene expression controlling cell expansion in a perennial grass species, tall fescue (*Festuca arundinacea* Schreb.). Tall fescue is a widely-used forage and turf grass and known for superior drought avoidance due to deep rooting characteristics (Qian et al., 1997; Huang and Fry, 1998; Huang and Gao, 2000). Understanding how antioxidants and oxidative defense may be involved in root responses to water stress would provide further insights into strategies for genetic modification or chemical-priming to promote root growth of plants that exposed to prolonged periods of water stress in water-limiting environments.

## Materials and methods

### Plant materials and growth conditions

The experiment was conducted in a hydroponic system for convenience of non-destructive monitoring of root growth and minimizing damages of roots during root treatment and sampling. Seedlings of tall fescue “K-31” were initially established from seeds planted in fritted clay medium (Profile Products, Deerfield, IL) for about 3 weeks. Seedlings of similar sizes and developmental stages were then carefully rinsed with water and roots were washed free of soil medium, and transferred to the hydroponic growing medium. About three to five seedlings were wrapped together at the base part of tillers using a foam cube, inserted in a polystyrene panel which was pre-drilled with 9 × 7 holes, and floated in a plastic container (50.8 cm × 36.8 cm × 15.2 cm) filled with half-strength Hoagland's nutrition solution (Hoagland and Arnon, 1950). To ensure the appropriate aeration, the nutrition solution was continuously aerated using an air pump. The experiment was carried out in a controlled environment room (Environmental Growth Chamber, Chagrin Falls, Ohio), which was set to maintain 22/18°C (day/night), 60% relative humidity, 12-h photoperiod, and 650 μmol m^−2^ s^−1^ photosynthetically active radiation (PAR) at the canopy level.

### Treatments and experimental design

Seedlings of 3-week-old were cultured in the half-strength Hoagland's nutrition solution for additional 7 days before exposure to water stress. Water stress was induced by adding polyethylene glycol (PEG)-8000 with incremental concentration into the nutrition solution which is a widely used osmotic regulator in hydroponic system imposing water deficit in plants (Lagerwerff et al., 1961; Janes, 1974). The osmotic potential of the solution was first adjusted to −0.25 MPa with PEG for 3 days, and then more PEG was added to bring the osmotic potential to −0.5 MPa to induce water stress. The non-stress control plants were grown in the half-strength Hoagland's nutrition solution without adding PEG.

For ascorbate (ASA) treatment, root systems of seedlings with or without PEG treatments were first incubated in half-strength Hoagland's nutrition solution without ASA (ASA-untreated control) or containing 5 mM ASA for 10 h (ASA treatment), and then transferred back into the original growth conditions as described above. The optimal concentration of ASA used here was chosen after a preliminary experiment with 0.25, 0.5, 1, 5, and 10 mM ASA, which has showed that 5 mM was the most effective in improving root growth.

Water stress and ASA treatment were arranged in a split-plot design with four containers of plants (or four replicates) exposed to either water stress or non-stress condition as main plots, and approximately 120 plants with either ASA-treated or -untreated plants randomly placed inside each water-stress or non-stress container as subplots.

### Analysis of root elongation rate

Newly-formed roots approximately 1-cm long were selected from 15 plants in each replicate or container for water stress or ASA treatment for the analysis of root elongation rate. Roots attached to the plant were carefully taken out of the culture solution, the length of roots was measured quickly using a plastic ruler, and then the plants were returned to the culture solution. Root length was measured daily for a period of 7 days. Root elongation rate was calculated as the daily average increase of root length per root.

### Analysis of malondialdehyde (MDA) content in roots

Malondialdehyde is the final product of lipid peroxidation in plant tissues and was quantified according to the procedure described by Zhang and Kirkham (1996) with slight modifications. The MDA content was measured for tissues from the root elongation zone (apical 1 cm) and for the maturation zone (basipetal 1–5 cm). Root tissues (0.5 g) of the tip or the base were washed free of culture solution and ground to powder in liquid nitrogen, and then homogenized in 6 mL 0.1% trichloroacetic acid (TCA). The homogenate was centrifuged at 10,000 g for 10 min, and 1 mL supernatant was added to 4 mL 10% TCA containing 0.5% thiobarbituric acid. The mixture was incubated at 95°C for 30 min, quickly cooled on ice, and centrifuged at 10,000 g for 10 min at 4°C. The absorbance of supernatant was measured at 532 and 600 nm using a spectrophotometer (Spectronic Instruments, Rochester, NY). The concentration of MDA was calculated using an extinction coefficient of 155 mM^−1^ cm^−1^ (Heath and Packer, 1968).

### Histochemical staining for hydrogen peroxide and superoxide in roots

Histochemical staining for the presence of hydrogen peroxide and superoxide was performed following treatments according to the procedures described in Thordal-Christensen et al. (1997) and Dunand et al. (2007), respectively, with slight modifications for each of them. To evaluate the presence of H_2_O_2_, roots were stained with 1% (w/v) 3-diaminobenzinidine (DAB; pH 3.8) for 1 h and subsequently rinsed with deionized water. To evaluate the presence of O${}_{2}^{-}$, roots were stained with 2 mM nitroblue tetrozolium (NBT) in 20 mM phosphate-buffered saline (PBS; pH 6.8) for 15 min and subsequently rinsed with deionized water. DAB- or NBT-stained roots were visually observed using an Olympus FSX100 Bio-imaging navigator (Olympus America, Central Valley, PA) and pictures were captured using bright-field single-shot mode at 4.2x magnification.

### Quantification of reactive oxygen species in roots

The production rate of O${}_{2}^{-}$ and content of H_2_O_2_ were measured for tissues from the root elongation zone (apical 1 cm) and for the maturation zone (basipetal 1–5 cm). The production rate of O${}_{2}^{-}$ was measured according to the procedure described by Bian and Jiang (2009) with slight modifications. Root tissues (0.2 g) were ground to powder in liquid nitrogen, homogenized in 1 mL 50 mM Tris-HCl (pH 7.5), and centrifuged at 5000 g for 10 min at 4°C. 200 μL supernatant was added to 800 μL 0.5 mM 3-bis (2-methoxy-4-nitro-5-sulfophenyl)-2H-tetrozolium-5-carboxanilide inner salt (XTT). XTT reduction was recorded once per minute for 3 min at 470 nm and the background absorbance was corrected with 50 units of superoxide dismutase (SOD). The O${}_{2}^{-}$ production rate was calculated using a 2.16 × 10^4^ M^−1^ cm^−1^ extinction coefficient and expressed as μmol O${}_{2}^{-}$ min^−1^ g^−1^ dry weight (DW) (Sutherland and Learmonth, 1997).

The content of H_2_O_2_ was measured according to the procedure described by Zhou et al. (2006) with slight modifications. Ground root tissues (0.5 g) were homogenized in 5 mL 5% (w/v) TCA and the homogenate was centrifuged at 10,000 g for 20 min at 4°C. The supernatant was adjusted to pH 8.4 with 17 M ammonia solution, briefly centrifuged to remove large particles, and divided into 1 mL aliquots. 8 μg catalase was added to one aliquot to serve as the blank. 1 mL colorimetric reagent solution containing 10 mg 4-aminoantipyrine, 10 mg phenol, and 5 mg peroxidase in 100 mM acetic acid buffer (pH 5.6) was added to each aliquot and the color reaction was incubated for 10 min at 30°C. Following incubation, the absorbance was measured at 505 nm and H_2_O_2_ content determined based on standard curve generated with known H_2_O_2_ concentrations.

### Quantification of non-enzymatic antioxidant content in roots

Endogenous content of free ascorbate and total ascorbate content were quantified for tissues from the root elongation zone (apical 1 cm) and for the maturation zone (basipetal 1–5 cm) according to the procedure described in Ma et al. (2008) with slight modifications. Frozen root powder (0.5 g) was homogenized in 8 ml 5% (w/v) TCA on ice, centrifuged at 10,000 g for 10 min at 4°C, and the supernatant was used immediately for analysis. For total ascorbate quantification, the supernatant was incubated in 200 mM PBS (pH 7.4) and 1.5 mM dithiothreitol (DTT) for 50 min to reduce all dehydroascorbic acid to ASA. Following incubation, 200 μL 0.5% (w/v) N-ethylmaleimide (NEM) was added to remove excess DTT. The resulting solution (0.8 ml) was then added to a reaction mixture containing 1 mL 10% (w/v) TCA, 800 μL 42% (w/v) o-phosphoric acid, 800 μL 65 mM 2,2′-dipyridyl in 70% (v/v) ethanol, and 400 μL 3% (w/v) iron (III) chloride. The reaction was incubated at 42°C for 1 h, and the absorbance was measured at 525 nm. Free ascorbate was measured using the procedure described above, while DTT and NEM were substituted with 400 μL deionized water. Free and total ascorbate content were determined based on a standard curve generated with known ASA concentrations.

### Quantification of enzymatic antioxidant activity in roots

Enzyme activities of CAT, POD, SOD, APX, DR, MR, and GR were measured for tissues from the root elongation zone (apical 1 cm) and for the maturation zone (basipetal 1–5 cm) according to the procedures described by Zhang and Kirkham (1996). For CAT, POD, and SOD assays, 0.5 g of root tissues were homogenized in 6 ml 50 mM PBS (pH 7.0) containing 0.2 mM ethylenediaminetetraacetic acid (EDTA) and 1% (w/v) polyvinylpyrrolidone (PVP) on ice, and the homogenates were centrifuged at 15,000 g for 20 min at 4°C. The absorbances were measured at 240, 470, and 560 nm for CAT, POD, and SOD, respectively. For APX, DR, MR, and GR assays, 0.5 g ground root tissues were homogenized in 6 ml 25 mM PBS (pH 7.8) containing 0.2 mM EDTA and 1% (w/v) PVP, and the homogenates were centrifuged at 15,000 g for 20 min at 4°C. The absorbance was measured at 290, 265, 340, and 340 nm for APX, DR, MR, and GR, respectively.

### Gene expression analysis of enzymatic antioxidants in roots

Gene expression analysis in the root elongation zone (apical 1 cm) and for the maturation zone (basipetal 1–5 cm) was performed by quantitative reverse transcriptase polymerase chain reaction (qRT-PCR). Total RNA was isolated from root tissue using TRIzol reagent (Life Technologies, Grand Island, NY) and treated with DNase (TURBO DNA-free kit; Life Technologies, Grand Island, NY) to remove contaminating genomic DNA. After that, 2 μg total RNA was reverse-transcribed using a high-capacity cDNA reverse transcription kit (Life Technologies, Grand Island, NY) and the synthesized cDNA was amplified in a StepOnePlus Real-Time PCR system (Life Technologies, Grand Island, NY) using the following parameters: pre-heat cycle of 95°C for 3 min, 40 cycles of 95°C denaturation for 30 s, and 60°C annealing/extension for 60 s. Power SYBR Green PCR Master Mix (Life Technologies, Grand Island, NY) was the intercalating dye used to detect gene expression level. Gene names, accession numbers, forward, and reverse primer sequences are provided in Table 1. A melting curve analysis was performed for each primer pair to confirm its binding specificity. *Actin* was used as the reference gene, since its expression was constant throughout treatments. A ΔΔCt method was used to calculate the relative expression level between genes of interest and reference gene, respectively.

**Table 1:** **Primer sequences used in qRT-PCR**.

**Gene**	**Accession number**	**Best BLAST hit**	***E*-value**	**Identity (%)**		**Primer sequence (5′–3′)**	**Tm (°C)**	**Size (bp)**
*CuZn-SOD*	DT712833.1	XM_003562436.2 (*Brachypodium distachyon*)	0	93	Forward	TATCCCCCTTACTGGACCACAT	61.7	85
					Reverse	GTGTCCACCCTTGCCAAGAT	61.6	
*Mn-SOD*	DT694762.1	XM_010233228.1 (*Brachypodium distachyon*)	1e-162	91	Forward	GGGCGCCATCAAGTTCAA	59.6	85
					Reverse	ACCCCCACCCTCATTAGCA	62.1	
*POD*	GT036635.1	XM_003566650.2 (*Brachypodium distachyon*)	0	90	Forward	CACATGCCCACAAGCTGATG	60.8	85
					Reverse	CAGAAGCGAAGCGGCAAT	59.8	
*CAT-A*	DT680104.1	XM_003573193.2 (*Brachypodium distachyon*)	0	90	Forward	CTGCTGGGCAACAACTTC	57.8	89
					Reverse	GACTTTGGGTTGGGCTTG	57.6	
*CAT-B*	DT704412.1	NM_001065170.1 (*Oryza sativa*)	2e-171	87	Forward	TCCTACGCTGATACCCAAAG	58.1	93
					Reverse	GTGATGGTTGTTGTGGTGAG	58	
*CAT-C*	AJ634002.1	XM_003558844.2 (*Brachypodium distachyon*)	0	91	Forward	GACCCACATCCAGGAGAAC	58.5	85
					Reverse	GTCGAAGAGGAAGGTGAACA	58.4	
*APX2*	DT702685.1	XM_010235217.1 (*Brachypodium distachyon*)	0	93	Forward	TTTGAGCGACCAGGACATTG	59.6	85
					Reverse	GGCTCCCTCAAAGCCAGATC	61.6	
*APX4*	DT714958.1	XM_003574845.2 (*Brachypodium distachyon*)	0	91	Forward	TGGTTTTGAAGGTGCATGGA	59.5	85
					Reverse	CCCCTCAGATTCTCCCTTCAG	60.5	
*DR*	DT684182.1	XM_003568966.2 (*Brachypodium distachyon*)	0	93	Forward	GTCACCCCTCCTGAGTATGCA	58.2	80
					Reverse	GTGGCATCCTTGCTCTTCAAG	57.8	
*GR*	GT036447.1	XM_003558703.2 (*Brachypodium distachyon*)	0	93	Forward	GCTGCACTAGACCTGCCTTCA	63.3	80
					Reverse	ATGCCAGCAAACTCCAAAGC	60.8	
*EXP-A3*	DT686661.1	AY692477.1 (*Triticum aestivum*)	4e-112	91	Forward	TGCCGTGCCGGAAGTC	61	72
					Reverse	TGATCAGCACCAGGTTGAAGTAG	61.4	
*XET1*	DT683504.1	AJ295943.1 (*Festuca pratensis*)	1e-173	92	Forward	GCACCGTCACAGCCTACTACCT	64.8	80
					Reverse	GGTCTCGTTGCCCAGGAA	60.7	
*XET2*	DT707331.1	AJ295944.1 (*Festuca pratensis*)	0	99	Forward	GCCCTACGTGATGAACACCAA	61.6	80
					Reverse	AGGGATCGAACCAGAGGTAGAAC	62.3	
*XET3*	AJ295945.1	Self			Forward	CGTTGATTCCGGTGCTAGCT	61.4	80
					Reverse	GTCGCAATCGTCGTTGAAGTT	60.4	
*Actin*	AY194227.1	Self			Forward	TCTTACCGAGAGAGGTTACTCC	59.3	107
					Reverse	CCAGCTCCTGTTCATAGTCAAG	59.5	

*Proposed gene names, GenBank accession numbers, best BLAST hit names, E-values, sequence identity scores, melting temperatures, and amplicon sizes are also listed*.

### Statistical analysis

Effects of water stress and ASA treatments on all parameters were determined using the Two-Way analysis of variance (ANOVA) using a statistical program (JMP11, SAS Institute, Cary, NC). Differences between mean values for each parameter were distinguished by student's *t*-test at the 0.05 probability level.

## Results

### Root elongation rate and membrane lipid peroxidation as affected by water stress alone and with additional ASA treatment

**Figure 1:**
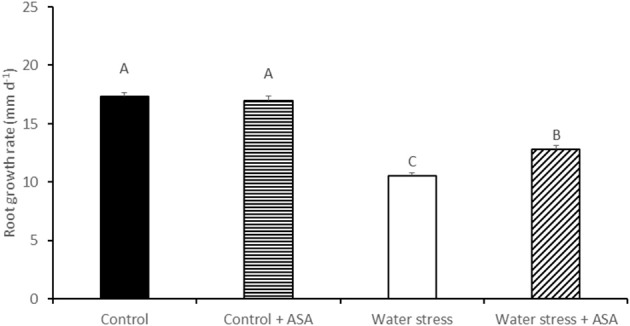
**Root growth rate of tall fescue exposed to non-stress control, non-stress control with ascorbic acid (ASA) treatment, water stress, and water stress with ASA treatment**. The data represent mean ± SE (*n* = 15 plants). Columns marked with the same letter are not significantly different at *p* < 0.05.

**Figure 2:**
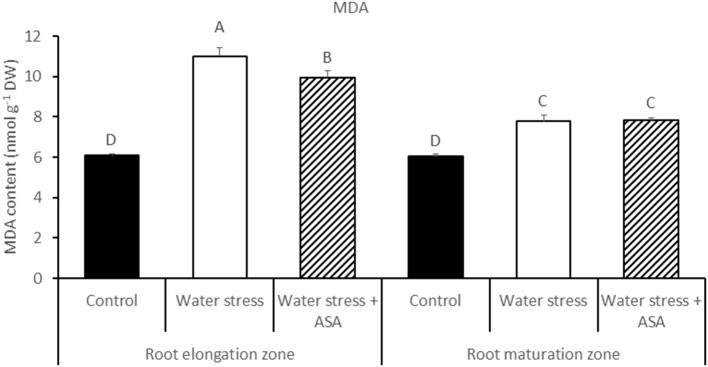
**The content of lipid peroxidation product (MDA) in tall fescue exposed to non-stress control, water stress, and water stress with ASA treatment**. The data represent mean ± SE (*n* = 4 replicated pots of plants and each pot with multiple plants). Columns marked with the same letter are not significantly different at *p* < 0.05.

Water stress induced by PEG caused significant reduction (by 59%) in root elongation rate, from 17.8 mm d^−1^ of the non-stress control plants to 10.6 mm d^−1^ of the water-stressed plants (Figure 1). The exogenous treatment of roots with ASA in the nutrition solution ameliorated the inhibitory effects of water stress on root elongation, causing a 72% increase in root elongation rate compared to ASA-untreated plants exposed to water stress (Figure 1). The exogenous treatment of roots with ASA under control condition did not have significant effect on root elongation rate. Further analysis of MDA content, ROS production, antioxidant enzyme activities, and gene expression were performed only in roots treated with or without ASA under water stress, but not conducted for roots treated with ASA under the non-stress control conditions due to the lack of ASA effects on root elongation under the non-stress conditions. The MDA content of both root elongation and maturation zones in PEG-treated plants was significantly (1.81-fold in elongation zone and 1.29-fold in maturation zone) higher than that that in the non-stress control, indicating the induction of oxidative damages in the entire roots by PEG-induced water stress (Figure 2). Exogenous ASA treatment resulted in significantly lower (1.64-fold) MDA content in the elongation zone of roots exposed to water stress, compared to the water stress treatment alone, but had no significant effects on MDA content of the maturation zone.

### Production of ROS in roots as affected by water stress alone and with additional ASA treatment

**Figure 3:**
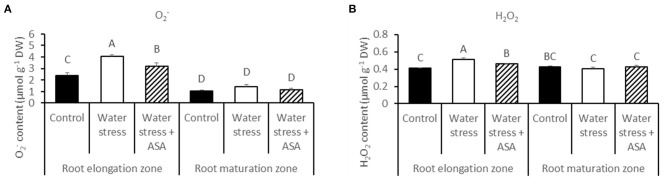
**The content of super oxide (O${}_{2}^{-}$) (A) and hydrogen peroxide (H_2_O_2_) (B) in roots of tall fescue exposed to non-stress control, water stress, and water stress with ASA treatment**. The data represent mean ± SE (*n* = 4 replicated pots of plants and each pot with multiple plants). Columns marked with the same letter are not significantly different at *p* < 0.05.

**Figure 4:**
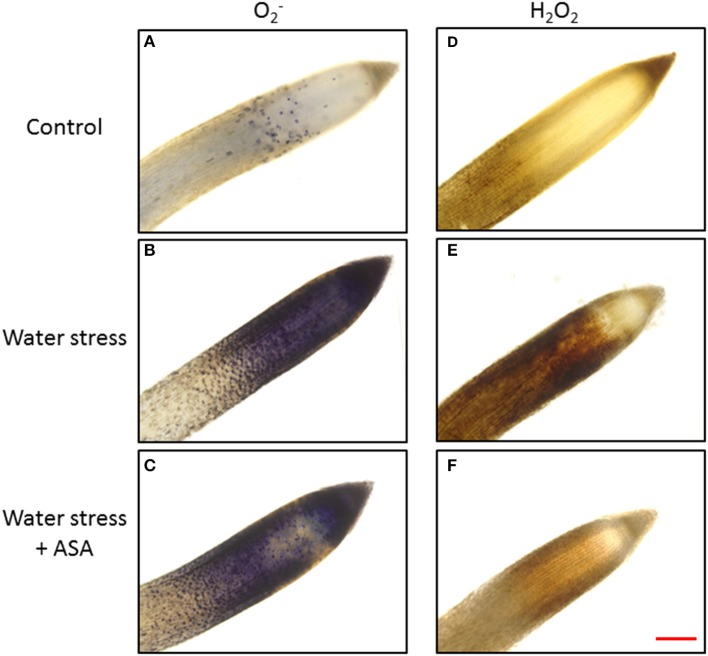
**Histochemical staining of tall fescue roots for visual localization of super oxide (O${}_{2}^{-}$) (A for non-stress control, B for water stress, and C for water stress with ASA treatment) and hydrogen peroxide (H_2_O_2_) (D for non-stress control, E for water stress, and F for water stress with ASA treatment)**. Bar represents for 100 μm.

The production of O${}_{2}^{-}$ and H_2_O_2_ was examined in both root elongation e and maturation zones using quantitative measurement (Figure 3) and histochemical staining (Figure 4) to determine level of oxidative stress induced by water stress and effectiveness of ASA treatment for oxidative scavenging in roots. Water stress significantly increased both O${}_{2}^{-}$ (1.68-fold) and H_2_O_2_ content (1.25-fold) in the root elongation zone, while it did not alter either O${}_{2}^{-}$ or H_2_O_2_ production in the maturation zone (Figures 3A,B). The histochemical staining also showed increased staining density for both O${}_{2}^{-}$ and H_2_O_2_ in the root elongation zone under water stress (Figures 4B,E). The ASA treatment caused significantly lower O${}_{2}^{-}$ (1.32-fold) and H_2_O_2_ (1.13-fold) content in the elongation zone than those without ASA treatment under water stress, but had not effects on either O${}_{2}^{-}$ or H_2_O_2_ in the maturation zone (Figures 3A,B). The histochemical staining pattern for ASA-treated roots also have less staining density for both O${}_{2}^{-}$ and H_2_O_2_ in the elongation zone, indicating ASA-mitigation of oxidative stress in the root elongation zone (Figures 4C,F).

### Production of antioxidant compounds in roots as affected by water stress alone and with additional ASA treatment

**Figure 5:**
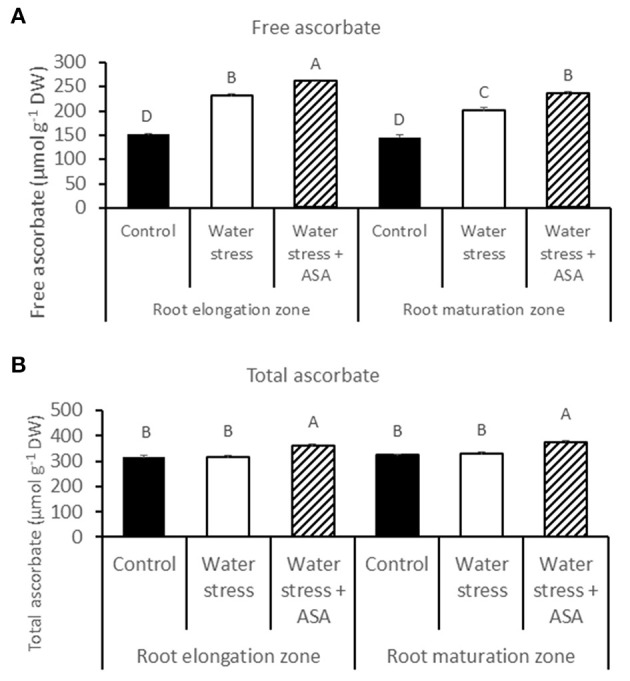
**Free (A) and total (B) ascorbate content in tall fescue roots exposed to non-stress control, water stress, and water stress with ASA treatment**. The data represent mean ± SE (*n* = 4 replicated pots of plants and each pot with multiple plants). Columns marked with the same letter are not significantly different at *p* < 0.05.

The endogenous content of free ascorbate and total ascorbate was quantified in both root elongation and maturation zones in order to evaluate whether ASA-mitigation of oxidative damages in roots due to water stress was associated with changes in the non-enzymatic antioxidant production. The content of free ascorbate increased under water stress in both root elongation and maturation zones, with 1.53- and 1.38-fold greater than the non-stress control, respectively (Figure 5). The exogenous ASA treatment caused significant higher content of endogenous free ascorbate, with 1.72-fold increase for the root elongation zone and 1.61-fold increase in the maturation zone under water stress (Figure 5A). The content of the total ascorbate content did not change under water stress, while exogenous ASA treatment significantly increased the total ascorbate content in both root elongation and maturation zones under water stress (Figure 5B).

### Antioxidant enzyme activities and transcript levels as affected by water stress alone and with additional ASA treatment

**Figure 6:**
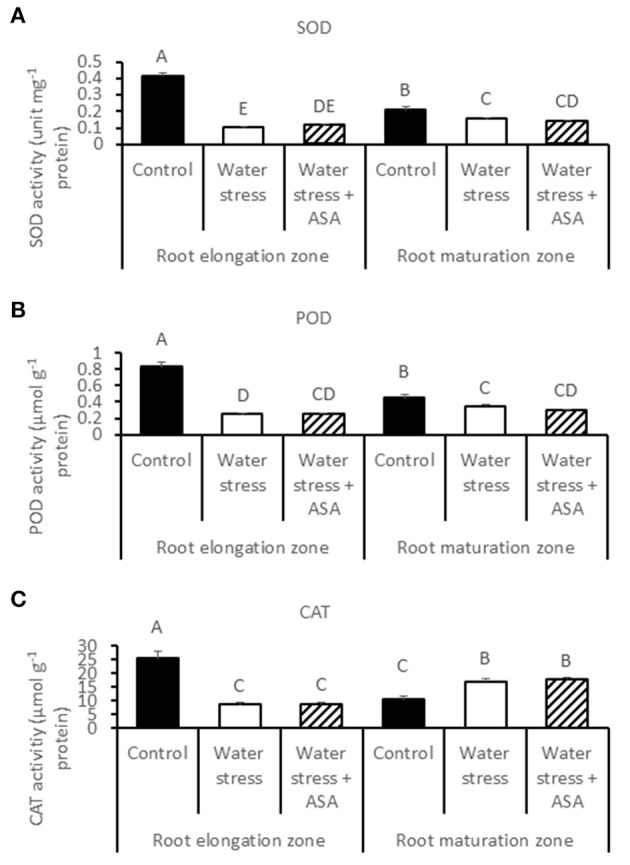
**Activity of superoxide dismutase (SOD) (A), peroxidase (POD) (B), and catalase (CAT) (C) in tall fescue roots exposed to non-stress control, water stress, and water stress with ASA treatment**. The data represent mean ± SE (*n* = 4 replicated pots of plants and each pot with multiple plants). Columns marked with the same letter are not significantly different at *p* < 0.05.

**Figure 7:**
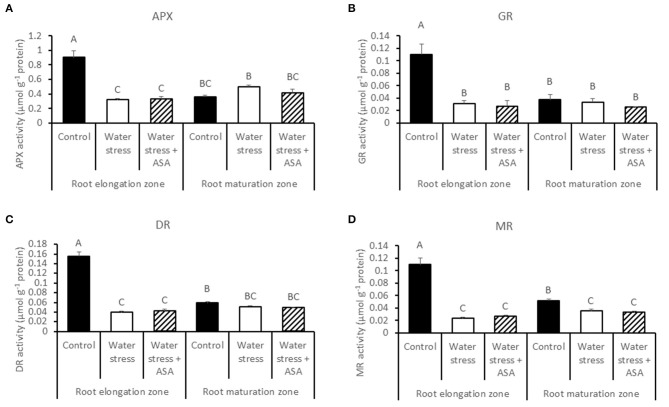
**Activity of ascorbate peroxidase (APX) (A), glutathione reductase (GR) (B), dehydroascorbate reductase (DR) (C), and monodehydroascorbate reductase (MR) (D) in tall fescue roots exposed to non-stress control, water stress, and water stress with ASA treatment**. The data represent mean ± SE (*n* = 4 replicated pots of plants and each pot with multiple plants). Columns marked with the same letter are not significantly different at *p* < 0.05.

ROS-scavenging enzyme activities and their respective transcript levels were examined in order to evaluate whether ASA-mitigation of oxidative damages in roots due to water stress was associated with the enzymatic ROS-scavenging system. The activities of all antioxidant enzymes examined in this study (SOD, POD, CAT, APX, GR, MR, and DR) decreased significantly in the root elongation zone under water stress compared to the non-stress control (Figures 6, 7). The activities of SOD, POD, and MR in the root maturation zone also decreased under water stress, but those of other enzymes in the root maturation zone did not differ between the non-stress control and water stress. Exogenous ASA treatment had no significant effects on the activities of antioxidant enzymes in ether root elongation or maturation zone.

**Figure 8:**
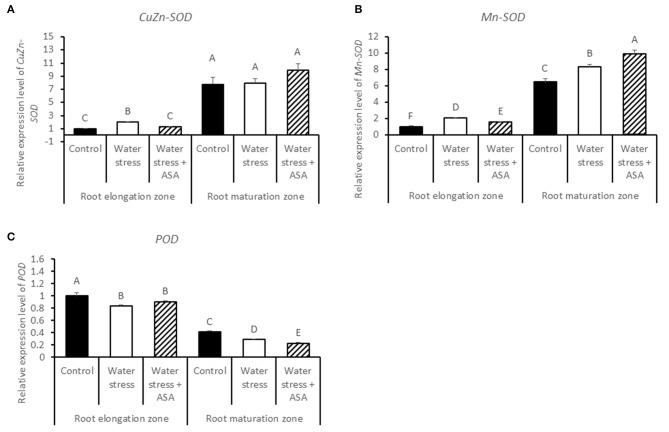
**Relative gene expression level of ***CuZn-SOD*** (A), ***Mn-SOD*** (B), and ***POD*** (C) in tall fescue roots exposed to non-stress control, water stress, and water stress with ASA treatment**. The data represent mean ± SE (*n* = 4 replicated pots of plants and each pot with multiple plants). Columns marked with the same letter are not significantly different at *p* < 0.05.

The transcript abundances of ROS-scavenging enzymes and cell-expansion-related proteins were detected by qRT-PCR. The *CuZn-SOD* and *Mn-SOD* transcript level in the root elongation zone were significantly higher in roots exposed to water stress with or without exogenous ASA treatment (Figures 8A,B). The transcript level of *CuZn-SOD* in the root maturation zone was not affected by either water stress or ASA treatment while *Mn-SOD* transcript level significantly increased under water stress without ASA treatment (1.28-fold) or with ASA (1.52-fold), and was significantly higher for ASA-treated than untreated roots exposed to water stress (Figure 8B). The *POD* transcript level was significantly decreased in both root elongation and maturation zones under water stress with or without ASA treatment (Figure 8C).

**Figure 9:**
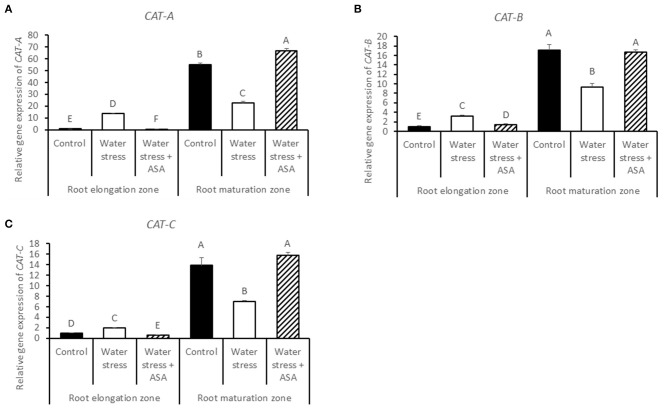
**Relative gene expression level of ***CAT-A*** (A), ***CAT-B*** (B), and ***CAT-C*** (C) in tall fescue roots exposed to non-stress control, water stress, and water stress with ASA treatment**. The data represent mean ± SE (*n* = 4 replicated pots of plants and each pot with multiple plants). Columns marked with the same letter are not significantly different at *p* < 0.05.

The transcript levels of *CAT-A, CAT-B*, and *CAT-C* in the root elongation zone were all significantly increased under water stress alone, which were significantly higher than the non-stress control or water stress with ASA treatment. The transcript levels of *CAT-A, CAT-B*, and *CAT-C* in the root maturation zone were all significantly decreased under water stress without ASA treatment while ASA treatment increased the transcript levels compared to the untreated plants (Figures 9A–C).

**Figure 10:**
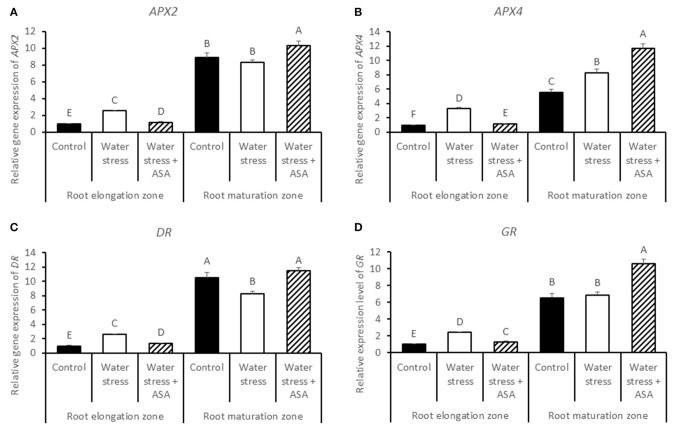
**Relative gene expression level of ***APX2*** (A), ***APX4*** (B), ***DR*** (C), and ***GR*** (D) in tall fescue roots exposed to non-stress control, water stress, and water stress with ASA treatment**. The data represent mean ± SE (*n* = 4 replicated pots of plants and each pot with multiple plants). Columns marked with the same letter are not significantly different at *p* < 0.05.

Transcript levels of *APX2* (Figure 10A), *APX4* (Figure 10B), *DR* (Figure 10C), and *GR* (Figure 10D) in the root elongation zone were significantly higher under water stress alone than those of the non-stress control or water stress with ASA treatment. Transcript levels of all four genes in the maturation zone were significantly greater in roots with ASA treatment than under water stress alone.

**Figure 11:**
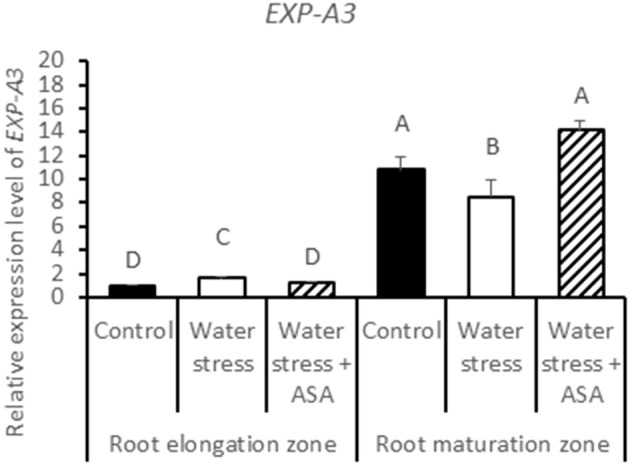
**Relative gene expression level of ***EXP-A3*** in tall fescue roots exposed to non-stress control, water stress, and water stress with ASA treatment**. The data represent mean ± SE (*n* = 4 replicated pots of plants and each pot with multiple plants). Columns marked with the same letter are not significantly different at *p* < 0.05.

**Figure 12:**
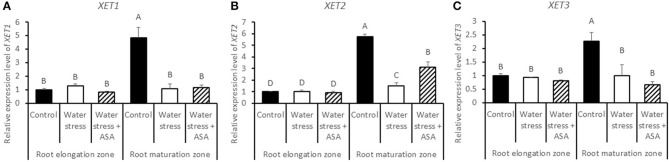
**Relative gene expression level of ***XET1*** (A), ***XET2*** (B), and ***XET3*** (C) in tall fescue roots exposed to non-stress control, water stress, and water stress with ASA treatment**. The data represent mean ± SE (*n* = 4 replicated pots of plants and each pot with multiple plants). Columns marked with the same letter are not significantly different at *p* < 0.05.

The expression levels of five expansin genes (*EXP-A3, EXP-A4, EXP-A5, EXP-B4*, and *EXP-B7*) and three XET genes (*XET1, XET2, and XET3*) regulating cell-wall loosening that were available from the tall fescue EST database, were examined to determine whether effects of water stress and ASA treatment on root elongation involve changes in different *expansin* and *XET* genes. Among five expansin genes, only *EXP-A3* exhibited differential expression in the root elongation and maturation zones among the non-stress control water stress and ASA treatment (Figure 11), while others did not show clear patterns in response to either water stress or ASA treatment in both root elongation and maturation zones (data not shown). Transcript level of *EXP-A3* significantly increased in the root elongation zone, but decreased in the maturation zone under water stress alone, while ASA treatment reversed *EXP-A3* responses to water stress, with a reduction and increase in the transcript level in the root elongation zone and maturation zone, respectively, compared to water stress alone (Figure 11). *XET1, XET2*, and *XET3* transcript levels in the root elongation zone were not significantly changed with water stress or ASA treatment (Figures 12A–C). In the root maturation zone, water stress led to significant decrease for *XET1* (22.0%), *XET2* (26.0%), and *XET3* (43.5%); exogenous ASA treatment of roots under water stress increased the transcript level of XET2 compared to with water stress alone, although it did not have significant effects on *XET1* and *XET3* (Figures 12A,C).

## Discussion

Cell growth is one of the most sensitive responses to water stress (Nilsen and Orcutt, 1996). Many metabolic factors could be involved in growth inhibition by water stress, such as excessive production of ROS, as mostly found that limit leaf growth (Hernández and Almansa, 2002; Nayyar and Gupta, 2006). The beneficial effects on plant survival rate, biomass, shoot, and root growth by exogenous ASA have been reported under either water (Shalata and Neumann, 2001; Athar et al., 2008) or heat stress (Kumar et al., 2011). In this study, root growth inhibition by water stress in tall fescue was accompanied by increasing O${}_{2}^{-}$ and H_2_O_2_ production mainly in the root elongation zone, which could lead to the increases in MDA content that reflect the extent of oxidative damages of cellular membranes. Our data is in accordance with previous research results in alfalfa (*Medicago sativa*, Wang et al., 2009), thale cress (*Arabidopsis thaliana*, Duan et al., 2010), and maize (*Zea mays*, Zhu et al., 2007; Yamaguchi and Sharp, 2010) that also showed ROS accumulation in association with root elongation inhibition under water stress. Antioxidants such as ASA, which are found at high concentrations mainly in chloroplasts in leaf tissues and other cellular compartments in roots, are known to play important roles in cellular defense against oxidative stress (Noctor and Foyer, 1998). Unlike more extensive studies in ASA protecting leaves from oxidative damages in, only a few reports of exogenous ASA application and its effect on root tissues were documented, either under non-stressed (Hidalgo et al., 1991; Cordoba-Pedregosa et al., 1996; Aroca, 2006; Tyburski et al., 2006), or salt stress condition (Shalata and Neumann, 2001; Afzal et al., 2006). In this study, exogenous ASA treatment mitigated root growth decline due to water stress, although it could not completely reverse the adverse effects of water stress. In addition, ASA treatment effectively suppressed the production of both O${}_{2}^{-}$ and H_2_O_2_, as well as MDA accumulation in the root elongation zone. These results suggested that the inhibitory effects of water stress on root elongation was associated with oxidative damages, which could be mediated by ASA mainly in the root elongation zone. The inhibitory effect of exogenous ASA on lipid peroxidation was also found in leaves (Zhang and Kirkham, 1996; Dolatabadian et al., 2008) and roots (Shalata and Neumann, 2001) in a few other plant species. The endogenous free and total ASA content indeed was increased with exogenous ASA treatment of roots exposed to water stress. The additional ASA could suppress ROS production and membrane lipid peroxidation serving directly as an antioxidant molecule, mitigating water stress-inhibition of root elongation in tall fescue.

Plant ROS scavenging system also involves the enzymatic pathways (Mittler, 2002). Water stress led to significant reduction in the activities of antioxidant enzymes, particularly in the root elongation zone, indicating that water stress weakened enzymatic antioxidant systems in roots, particularly in the root elongation zone, thereby inhibiting contribute to the inhibitory to root elongation. However, ASA mitigation of stress-inhibition of root elongation in tall fescue seemed not to be related to changes in the activities of antioxidant enzymes, as exogenous ASA treatment did not have significant effects on any of the enzymes in either root elongation zone or maturation zone. Therefore, water stress-induced oxidative stress in roots of tall fescue could be quenched directly by the non-enzymatic pathway without involving the enzymatic activities, since ASA is not only serves as the substrate for the enzymatic reaction catalyzing by APX, DR, or GR, but also is a strong reductant (Noctor and Foyer, 1998). In addition, exogenous application of ASA has been found to enhance cell proliferation in root primordia in several plant species (Citterio et al., 1994; de Cabo et al., 1996; Arrigoni et al., 1997), possibly due to their induced G1 to S progression in quiescent center cells (Liso et al., 1988; Navas and Gomez-Diaz, 1995). The transcript abundances of two antioxidant-enzyme genes, *CAT (-A, -B*, and *-C)* and *DR* in the root maturation zone decreased with water stress, but increased with exogenous ASA treatment in roots exposed to water stress, which corresponded to the water stress-inhibition and the ASA mitigation effects on root growth in tall fescue, suggesting the importance of CAT and DR could be transcriptionally involved in antioxidant protection from stress damages in root growth in tall fescue. The changes in the transcript levels of other antioxidant-enzyme genes exhibited variable responses to water stress and ASA in the root elongation or maturation zone. The enzymatic data and transcript levels are not always directly corresponded with each other, due to post-transcriptional and post-translational modifications, and due to the existence of multiple gene family for an enzyme protein. We have obtained all the transcript sequences available in GenBank database of tall fescue, but there may still be gene family members not discovered. For those reasons, the discrepancy between enzymatic activities and transcript levels was possible. However, the changes in transcript levels without changing enzymatic activities at least suggested transcriptional regulation of root growth could have occurred due to the application of ASA. The antioxidant mechanisms by which water stress and ASA affect root growth is complex and deserves further investigation.

Cell expansin and elongation are directly controlled by cell-wall loosening proteins, including expansins and XETs. Our data suggested that *EXP-A3* have increased transcript abundance in the root elongation zone, and decreased transcript abundance in the root maturation zone under water stress. Similarly, Wu et al. (2001) also reported that three expansins have increased transcript level in the apical region and decreased transcript level in the basal region of the root elongation zone under water stress. The transcript level of *XET2* was significantly decreased in the root maturation zone, corresponding with decreased root elongation rate under water stress. Furthermore, the effect of water stress on the transcript levels of *EXP-A3* and *XET2* could be reversed after the exogenous application of ASA, while the other expansin and XET family genes examined in this study exhibited variable responses to water stress and ASA in the root elongation or maturation zone. Nevertheless, these results indicated that *EXP-A3* and *XET2* could be involved in water stress-inhibition of root elongation and ASA-mediated promoting effects on root growth under water stress. Our results also suggested potential interactive roles of the two cell-wall loosening genes (*expansin* and *XET*) and antioxidant metabolism controlling root responses to water stress, but this postulation is worthy of further research.

In summary, tall fescue root growth could be enhanced by the exogenous application of ASA under water stress. The inhibition of root elongation by water stress was accompanied by increased production of O${}_{2}^{-}$ and H_2_O_2_, which was suppressed by the addition of ASA. The positive effects of ASA on root elongation under water stress were mainly due to the direct antioxidant effects of this strong reductant. The transcript levels of *CATs* and *DR* in root maturation zone were increased after ASA application under drought stress, compared with drought stress alone, although their enzymatic activities were not changed. Moreover, The *EXP-A3* and *XET2* transcript levels were also increased in root maturation zone with ASA treatment, which could be associated with the increased root growth under water stress. The direct association of *EXP*-*A3* and *XET2* with cell division and cell elongation controlling root elongation rate are unknown, which requires further investigation. This study also suggests potential interactions of antioxidant defense and cell-expansion genes controlling root elongation in responses to environmental stresses, which deserves further investigation.

### Conflict of interest statement

The authors declare that the research was conducted in the absence of any commercial or financial relationships that could be construed as a potential conflict of interest.

## Abbreviations

APX: ascorbate peroxidase
ASA: ascorbic acid
CAT: catalase
DR: dehydroascorbate reductase
EXP: expansin
GR: glutathione reductase
MDA: malondialdehyde
MR: monodehydroascorbate reductase
PBS: phosphate-buffered saline
PEG: polyethylene glycol
POD: peroxidase
qRT-PCR: quantitative reverse transcriptase polymerase chain reaction
ROS: reactive oxygen species
TCA: trichloroacetic acid
XET: xyloglucan endotransglycosylase.
